# Supplementary material for: The Small RNA Universe of Capitella teleta
Source: Front Mol Biosci. 2022 Feb 25;9:802814. doi: 10.3389/fmolb.2022.802814 (PMC8915122; doi:10.3389/fmolb.2022.802814)
Supplement: Supplementary file 1 [file DataSheet1.ZIP › Supplement/known/CAPTEscaffold_130_11169.pdf]

Provisional ID	:	CAPTEscaffold_130_11169
Score total	:	5474.8
Score for star read(s)	:	3.9
Score for read counts	:	5464.9
Score for mfe	:	1.4
Score for randfold	:	1.6
Score for cons. seed	:	3
Total read count	:	10731
Mature read count	:	10710
Loop read count	:	0
Star read count	:	21

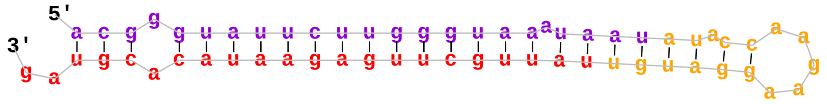

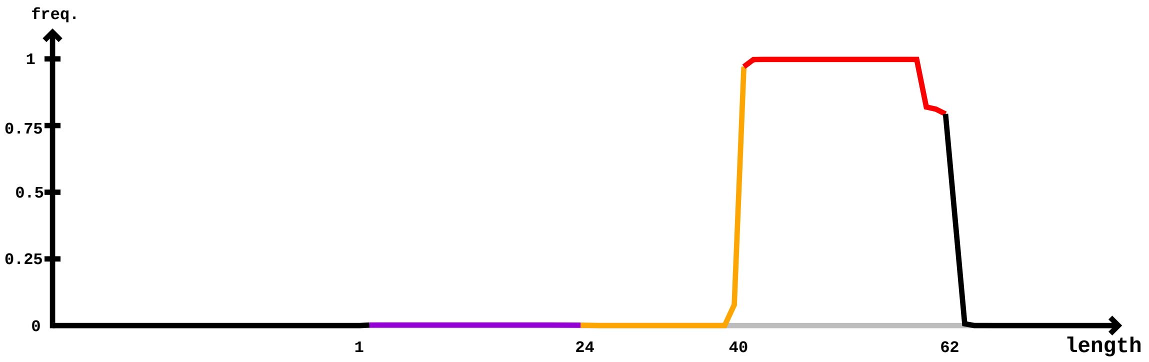

Star Mature

```
5'- cauuggcggaaagaagaacgcuagguugaguacggguauucuuggguaaauaauauaccaagaaggauguuauugcuugagaauacacguagcaaaccuaagcuuuauc   -3'   obs
    cauuggcggaaagaagaacgcuagguugaguacggguauucuuggguaaauaauauaccaagaaggauguuauugcuugagaauacacguagcaaaccuaagcuuuauc         exp
    cauuggcggaaagaagaacgcuagguugaguacggguauucuuggguaaauaauauaccaagaaggauguuauugcuugagaauacacguagcaaaccuaagcuuuauc         known
    .....(((..........)))((((((...((((.((((((((((((((.((((((.((.....)))))))))))))))))))))).))))...)))))).........   reads   mm   sample

    ...............................acggguauucuuggguaaau..........................................................   2       0    seq
    ...............................acggguauucuuggguaaaua.........................................................   2       0    seq
    ...............................acggguauucuuggguaaauaa........................................................   3       0    seq
    ...............................acggguaAucuuggguaaauaau.......................................................   1       1    seq
    ...............................acggguauucuuggguaaauaau.......................................................   11      0    seq
    ...............................acggguauucuuggguaaauaaua......................................................   1       0    seq
    ................................cggguauucuuggguaaauaaua......................................................   1       0    seq
    .....................................................uauaccaagaaggauguuauugcu................................   1       0    seq
    .....................................................................Cuauugcuugagaauacacg....................   1       1    seq
    .....................................................................uuauugcuugagaauacacg....................   98      0    seq
    .....................................................................uuauugGuugagaauacacgu...................   1       1    seq
    .....................................................................uuauugcuugagaauacacgu...................   77      0    seq
    .....................................................................uuauugcuugagaauacacguG..................   1       1    seq
    .....................................................................uuauugcuugagaauacacgua..................   140     0    seq
    .....................................................................uuauugAuugagaauacacgua..................   1       1    seq
    .....................................................................uuauugcuugagaauacacguGg.................   1       1    seq
    .....................................................................uuauugcuugagaauacacguaU.................   5       1    seq
    .....................................................................uuauAgcuugagaauacacguag.................   1       1    seq
    .....................................................................uuauugcuugagaauacacguaA.................   28      1    seq
    .....................................................................uuauugcuugagaauacacguag.................   469     0    seq
    .....................................................................uuauugcuugagaauacUcguag.................   1       1    seq
    .....................................................................Guauugcuugagaauacacguag.................   1       1    seq
    .....................................................................uuauugcuugagaauacacguagA................   10      1    seq
    ......................................................................uauugcuugagaauacaG.....................   1       1    seq
    ......................................................................uauugGuugagaauacacg....................   1       1    seq
    ......................................................................uauugcuugagaauacacg....................   1738    0    seq
    ......................................................................uauugcAugagaauacacg....................   1       1    seq
    ......................................................................uauuCcuugagaauacacg....................   2       1    seq
    ......................................................................Aauugcuugagaauacacg....................   2       1    seq
    ......................................................................uauugcuugaAaauacacg....................   1       1    seq
    ......................................................................uauugcuugagaauacUcg....................   1       1    seq
```

Star Mature

```
cauuggcggaaagaagaacgcuagguugaguacggguauucuuggguaaauaauauaccaagaaggauguuauugcuugagaauacacguagcaaaccuaagcuuuauc
......................................................................uaAugcuugagaauacacg....................  2     1  seq
......................................................................uauugAuugagaauacacg....................  1     1  seq
......................................................................uauugcuuAagaauacacg....................  5     1  seq
......................................................................Gauugcuugagaauacacg....................  1     1  seq
......................................................................uauugcuAgagaauacacg....................  2     1  seq
......................................................................uauugcuugagaauacGcg....................  1     1  seq
......................................................................uauugcuugagaauaUacg....................  1     1  seq
......................................................................uauugcuugagaauacacU....................  1     1  seq
......................................................................uauugcuugagaauacacA....................  4     1  seq
......................................................................uauugcuugagaauacacgu...................  8     0  seq
......................................................................uauugcuugagaauacacgua..................  51    0  seq
......................................................................uauugcuugagUauacacguag.................  1     1  seq
......................................................................uauugcuugaUaauacacguag.................  1     1  seq
......................................................................uauugcuugagaauacaGguag.................  2     1  seq
......................................................................uauugcuugagaauacacguUg.................  1     1  seq
......................................................................uauugcGugagaauacacguag.................  1     1  seq
......................................................................uauugcuugagaauaUacguag.................  3     1  seq
......................................................................uauugcuAgagaauacacguag.................  6     1  seq
......................................................................uaAugcuugagaauacacguag.................  3     1  seq
......................................................................uauugcuuAagaauacacguag.................  14    1  seq
......................................................................uauugcuugagaauGcacguag.................  3     1  seq
......................................................................uauugcuugagaauaAacguag.................  4     1  seq
......................................................................uauugcuugagaauacacAuag.................  1     1  seq
......................................................................uauugcuugagaGuacacguag.................  2     1  seq
......................................................................uaCugcuugagaauacacguag.................  1     1  seq
......................................................................uauugcuugagaauacacUuag.................  3     1  seq
......................................................................uauugcuCgagaauacacguag.................  1     1  seq
......................................................................uauugcuugagaauacGcguag.................  1     1  seq
......................................................................uaNugcuugagaauacacguag.................  1     1  seq
......................................................................uauugcCugagaauacacguag.................  2     1  seq
......................................................................uauugcuugagaauUcacguag.................  1     1  seq
......................................................................uauugcuugagGauacacguag.................  1     1  seq
......................................................................Aauugcuugagaauacacguag.................  21    1  seq
......................................................................uauugcAugagaauacacguag.................  2     1  seq
......................................................................uauugcuugagaauacacguaU.................  7     1  seq
......................................................................uauugcuugagaaCacacguag.................  1     1  seq
......................................................................uauuUcuugagaauacacguag.................  1     1  seq
......................................................................uauugcuugagaaAacacguag.................  2     1  seq
......................................................................uGuugcuugagaauacacguag.................  1     1  seq
......................................................................uauugAuugagaauacacguag.................  1     1  seq
......................................................................Cauugcuugagaauacacguag.................  1     1  seq
......................................................................uauugcuugagaauacacguGg.................  4     1  seq
......................................................................uauugcuugaAaauacacguag.................  1     1  seq
......................................................................uauugcuugagaauacUcguag.................  2     1  seq
......................................................................uauugcuugagaauacacguaA.................  13    1  seq
......................................................................Nauugcuugagaauacacguag.................  4     1  seq
......................................................................uaGugcuugagaauacacguag.................  1     1  seq
......................................................................uauAgcuugagaauacacguag.................  1     1  seq
......................................................................Gauugcuugagaauacacguag.................  4     1  seq
......................................................................uauugcuuCagaauacacguag.................  1     1  seq
......................................................................uauugcuugagaauacacguag.................  6997  0  seq
......................................................................uauugcuugagaauacacguagA................  347   1  seq
......................................................................uauugcuugagaauacacguagU................  226   1  seq
......................................................................uauugcuugagaauacacguagG................  5     1  seq
......................................................................uauugcuugagaauacacguagAa...............  61    1  seq
......................................................................uauugcuugagaauacacguagAaaa.............  1     1  seq
.......................................................................auugcuugagaauacacg....................  50    0  seq
.......................................................................auuAcuugagaauacacg....................  1     1  seq
.......................................................................auAgcuugagaauacacguag.................  1     1  seq
.......................................................................auugcuAgagaauacacguag.................  1     1  seq
.......................................................................auugcuGgagaauacacguag.................  1     1  seq
.......................................................................auugcuuAagaauacacguag.................  1     1  seq
.......................................................................auugcuugagaauacacguag.................  197   0  seq
.......................................................................Guugcuugagaauacacguag.................  1     1  seq
.......................................................................auugcuugagaauacacguagA................  8     1  seq
.......................................................................auugcuugagaauacacguagU................  23    1  seq
.......................................................................auugcuugagaauacacguagUa...............  1     1  seq
.......................................................................auugcuugagaauacacguagAa...............  2     1  seq
........................................................................uugcuugagaauacacguagAa...............  6     1  seq
```
